# On the Shaping of a Short Signal at the Output of the Receiving Piezoelectric Transducer in the Radiation-Reception System

**DOI:** 10.3390/ma11060974

**Published:** 2018-06-08

**Authors:** Boris Ee, Roman Konovalov, Sergey Konovalov, Andrey Kuz’menko, Valery Tsaplev

**Affiliations:** Department of Electroacoustics and Ultrasonic Engineering, Saint Petersburg Electrotechnical University “LETI”, Prof. Popov, 5, Saint Petersburg 197376, Russia; william47@mail.ru (B.E.); rskonovalov.eut@gmail.com (R.K.); agkuzmenko.eut@gmail.com (A.K.); valery@convergences-fr.ru (V.T.)

**Keywords:** piezoceramic plate, radiation-receiving system, principle d’Alembert, finite element method (FEM), piezoelectric transducer, acoustic pulse, electric pulse, compensating pulse

## Abstract

This paper theoretically and experimentally considers the pulsed mode of operation of the radiation-receiving system. The system contains two identical piezoceramic plates separated by a layer of immersion liquid (glycerin). The emitter was excited by the complex electrical signal of the special shape, which consisted of two half-cycles of the sine wave (exciting and compensating) on the natural frequency of the piezoplates. The forms of these signals were calculated by the authors and described in their previous papers using the d’Alembert method. The length of the electrical signal was estimated at the output of the piezoelectric receiver. The problem was solved theoretically using the finite element method. The acoustical system was simulated with the help of the COMSOL Multiphysics modeling environment. A comparative study of the theoretical and experimental results is carried out. The form of the signal at the output of the system was calculated by the d’Alembert method, and the simulated form by the finite element method was in good coincidence with the results of experimental and full-scale modeling. It is shown that the usage of complex waveforms allows achieving a significant pulse duration reduction of the electrical voltage at the output of the receiver.

## 1. Introduction

The internal structure of materials and products can be studied using various methods. One of the most common is the acoustic method, which has a number of inherent advantages. These include security, the possibility of carrying out measurements with unilateral access to the object of control, cheapness, etc. Technical tools that implement different approaches to the solution of a great number of control problems are quite various. What unites them is the presence of a constructive element intended for the radiation and reception of acoustic signals: the electroacoustic transducer. It can be based on different physical principles, but currently, the most common methods are based on piezoelectric transducers (PET). The use of thin-film piezoelectric transducers seems to be very promising [1,2]. The theoretical basics of these transducers are well described in the scientific and technical literature. Depending on the requirements that are imposed on the control and measuring equipment, there is a need to formulate the requirements of transducers. Considerations of the theory of their operation, design, and manufacturing technology have been the subject of many papers by various authors. It is not possible to provide a whole list of bibliographic data relating to this issue in one article. We can only mention, as an example, several works that have become classics [3,4,5,6,7,8,9,10,11,12,13,14,15].

Many ultrasonic measuring systems used in medical diagnostics, non-destructive testing, and hydroacoustics require the usage of piezoelectric transducers that have been especially designed for solving special problems that arise in the practice of ultrasonic testing. One of the most important problems facing the developers of transducers is the creation of PET with preset properties. For example, when solving the location problems, it is often necessary to have the transducer able to operate in the mode of short acoustic signals. In this case, it is necessary to use special methods to obtain the duration of the emitted acoustic pulse under the resonant excitation of the transducer, which must not exceed several half-periods of the main frequency. In order to solve this problem, the designer must provide in advance the possible ways of implementing the design of the PET, which need to be adapted in order to radiate the signal of the desired length.

Piezoelectric transducers that are capable of operating in the emission and reception modes of short signals can improve the most important characteristics of the control and measuring echolocation devices (resolution, the dead zone length, the positioning accuracy). The usage of such equipment is also relevant in problems related to thickness measurements, such as when the thickness of the product is very small compared with the spatial length of the probing pulse, as well as in cases where the test sample is a set of closely spaced layers.

Usually, a piezoelectric transducer must have a wide bandwidth to radiate a short acoustic signal. It is possible to achieve this goal by different methods [16,17,18]. The methods of mechanical damping, the application of matching layers, and the connection of electric-correcting circuits to the electrical output of the transducer are the most widespread nowadays. In this paper, we do not consider the problems of the expansion of the bandwidth of the PET to obtain short signals. 

Another possible way to get a short signal at the output of the transducer is the usage of narrow-band transducers. In this case, the effect of reducing the duration of the acoustic signal radiated by the transducer (for example, piezoelectric plate) is as follows. The active element of the transducer is excited by an electric signal with a complex shape. For example, such a signal may consist of two half-periods of the sine wave of the piezoelectric plate’s natural frequency. The first half-period excites the plate, and causes a rather long transition process in the transducer. This signal provides a long radiated acoustical pulse. The second half-cycle is the compensating one and it also causes a transition process. If the compensating pulse has a predetermined sign and amplitude, then one transient process will partially compensate the other. It is possible to adjust the duration of the radiated acoustic signal by choosing the initial time of the second half-period. The theoretical method, which enabled calculating the parameters of electric compensating pulses, is based on the application of the d’Alembert method [19,20]. A theoretical comparison of the acoustic signal at the output of the radiator by the d’Alembert method (not accounting for the direct piezoelectric effect) and the Fourier spectral method (taking it into account in combination with the method of equivalent circuits) was performed in Ref. [21]. Both results were similar, which indicated that the d’Alembert method is valid to assess the shape of signals radiated by the piezoelectric transducer. The problem was solved using the traditional approximation for plates. This means that the thickness is small in comparison with the transverse dimensions. This, in turn, means that the model is based on classical approaches (without taking into account spatial effects related to the limitations of transducers, diffraction phenomena, chaotic dynamics in transducers, etc.). This allowed most simply to identify the main mechanisms of the studied physical processes. The possibility of reducing the duration of the signal at the output of the receiver in the radiation-reception system (which consists of two piezoceramic plates separated by a layer of immersion liquid) were investigated theoretically and experimentally in Ref. [22]. It was shown that the calculated and the experimental data coincide well. It is of interest to continue the research initiated in the previous works of the authors. This work continues the research initiated in the previous studies of the authors.

As before, we still consider the resonant excitation of the piezoelectric plate. It should be added also that the studies described below were performed within the traditional frequency range of ultrasonic non-destructive testing, which is conditionally enclosed within the boundaries of 0.5–25 MHz.

Continuing on from earlier studies, it is useful to note once again that in [19,20,21], we studied the possibility of obtaining short acoustic pulses at the output of the radiating piezoelectric plate and an electric signal at the output of the radiating-receiving system (consisting of two plates) by the d’Alembert method (method of successive reflections). The radiating-receiving system consisted of two plates, and the one-dimensional case of radiation and reception of plane waves was considered. 

It is now interesting to consider the solution of the same problem using the finite element method. Currently, there is a large number of theoretical works devoted to the analysis of the operating modes of piezoelectric transducers. Among them, we can note, for example, [23,24,25,26,27,28,29]. The number of works devoted to the study of piezoelectric transducers in the various fields of modern acoustics (biomedical research, hydroacoustics, flaw detection, etc.) that are associated with the use of the finite element method is constantly growing. This confirms the relevance of this method. A comparison of theoretical and experimental results is also of interest. This paper is devoted to the study of these problems. 

## 2. Experimental Study of the Radiating-Receiving System

The paper presents the results of an experimental study of forming a short pulse at the output of the radiating-receiving system [22]. Here, we shall discuss the results achieved in a nutshell.

**Figure 1:**
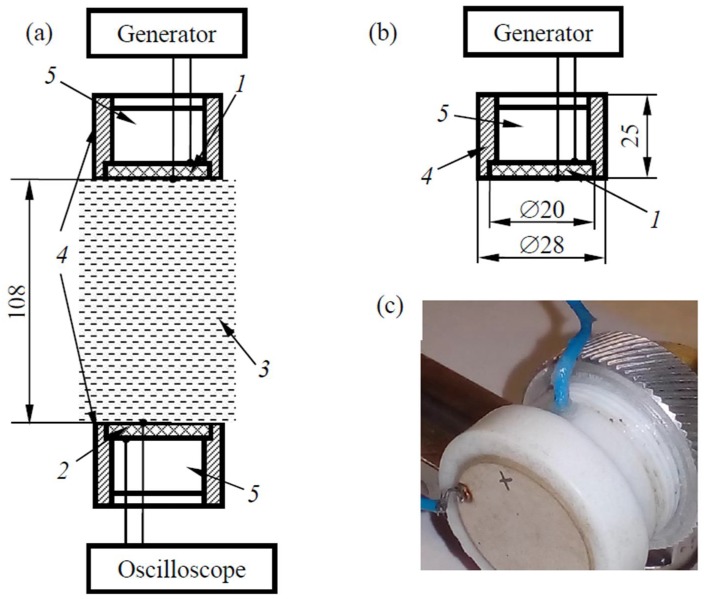
Experimental setup: (**a**) pattern, (**b**) diagram, and (**c**) photo of the piezoelectric transducer.

Experimental studies were carried out using a laboratory setup, which is shown in Figure 1a. The setup includes radiating 1 (Figure 1b,c) and receiving *2* piezoelectric transducers (two identical piezoelectric plates fabricated from the piezoceramics PZT-19, the properties of which are given in Section 3.2). The radiator was excited by a special form signal generator Tabor Electronics WW2572A. The signal at the output of the receiver was analyzed using an oscilloscope LECROY WaweAce 101. The electrical signals from the generator output that were fixed by the oscilloscope were processed digitally by the computer. Glycerin *3* was used as an immersion liquid (density 1260 kg/m^3^, sound velocity 1920 m/s), which eliminated the possibility of an electrical short. The parameters of piezoceramic plates were: main frequency—1 MHz, diameter—20 mm, thickness—1.25 mm. The back faces of both plates were loaded onto the air *5* inside the fluoroplastic cases 4.

The described radiating-receiving system had the ability to adjust the distance between the working faces of piezoelectric plates. This distance during the experimental measurements was equal to 108 mm. The alignment of the system was achieved by determining the position of the piezoelectric transducers, where the maximum signal was observed at the output of the receiver in a continuous mode of radiation.

**Figure 2:**
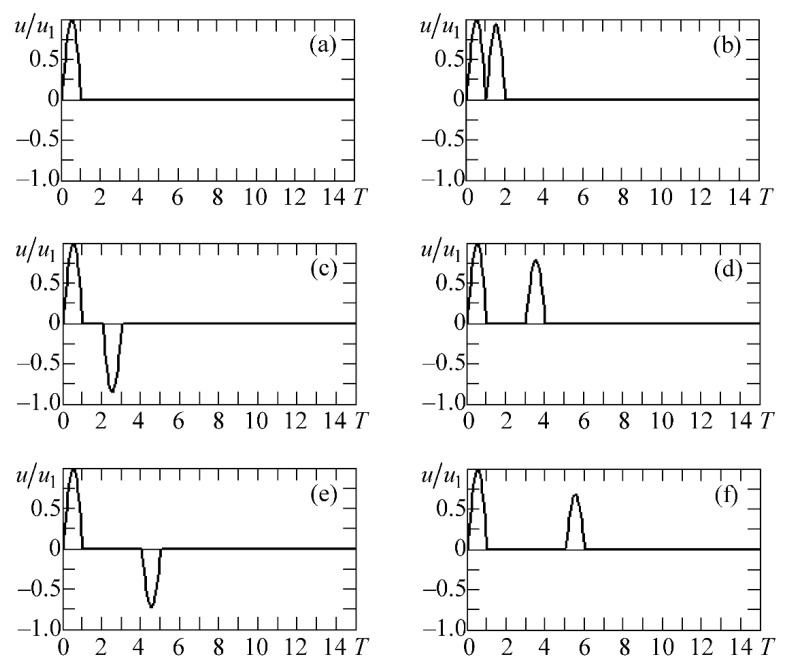
Shapes of electrical signals exciting the radiating piezoelectric plate: (**a**) shows the electric signal consisting of only one (exciting) half-period of the sine wave supplied to the radiator; (**b**–**f**) shows the electrical pulses (exciting and compensating half-waves).

The radiator was excited by an electric signal of a complex shape. The shape was calculated using the d’Alembert method on the basis of the algorithm described in [19,20]. The shapes of some signals are shown in Figure 2. The abscissa axis is for the dimensionless time $T=t/\left(T_{0}/2\right)$, where t—physical (true) time, $T_{0}$—is the period of oscillations at the anti-resonance frequency of the plate. The ordinate axes are for the electric voltage $u/u_{1}$ normalized to the amplitude *u*_1_ of the exciting half-wave. Figure 2a shows the electric signal consisting of only one (exciting) half-period of the sine wave supplied to the radiator. Figure 2b–f shows the electrical pulses of the complicated forms exciting the radiating plate. They contain exciting and compensating half-waves. The amplitudes of the exciting half-waves, due to the accepted normalization, are always equal to 1, and the amplitudes of the compensating half-waves are: 0.926 (Figure 2b); −0.852 (Figure 2c); 0.789 (Figure 2d); −0.726 (Figure 2e); and 0.672 (Figure 2f).

Electrical signals at the output of the receiver were displayed on the oscilloscope screen in a certain time range.

The shapes of electrical signals at the output of the radiation-receiving system, depending on the signals at the input of the radiator, are given below in comparison with the results of the solution obtained by the finite element method.

## 3. The Solution of the Problem by Finite Element Method

At the first stage of research, the problem of obtaining short signals at the output of the system was solved analytically by the method of successive reflections (the d’Alembert method) [19,20]. The application of this method allowed obtaining the estimated values of the required amplitudes of the exciting and compensating half-waves of the signal applied to the radiator. 

This problem was solved for a one-dimensional piezoelectric plate. Traditionally, it was admitted that its thickness was small compared to the size of the aperture. However, in real cases, it is necessary to take into account the limited size of the piezoelectric plate transducer. In these cases, the model becomes more complex, and the analytical solution becomes more difficult. It is more convenient to use numerical simulation methods, such as the finite element method (FEM), boundary element method (BEM), spectral element method (SEM), finite difference method (FDM), etc. We used the finite element method of the COMSOL Multiphysics modeling environment with the Structural Mechanics, Acoustics, and Electrostatics customized user modules.

### 3.1. Theoretical Background

Electrical and mechanical phenomena in the radiation-reception system (mechanical strain u, mechanical stress σ, electrical field strength E, and electrical displacement D) can be described using the pair of the fundamental equations of the direct and reverse piezoelectric effects:

$$\begin{array}{l} u_{i}=s_{ij}^{E}\sigma_{j}+d_{im}E_{m}\\ D_{m}=d_{mi}\sigma_{i}+\varepsilon_{mk}^{\sigma }E_{k} \end{array}\}$$

where $s_{ij}^{E}$ is the mechanical compliance of the material measured at zero electric field (E=0), $\varepsilon_{mk}^{\sigma }$ is the dielectric permittivity measured at zero mechanical stress (σ=0), and $d_{mi}$ is the piezoelectric modulus.

The acoustic wave propagation can be described by the wave equation:

(1) $$\frac{1}{c}\frac{d^{2}p}{dt^{2}}=\nabla \left(-\nabla p\right),$$

where *p*—sound pressure in liquid, ∇—Hamiltonian, and *c*—sound speed in the liquid.

The transfers of mechanical displacements and stresses from the piezoelectric radiator to the medium and from the medium to the receiving piezoelectric element are described by the following boundary condition: continuity of the normal component of the acceleration at the surface:

$$n\left(-\nabla p\right)=n\frac{d^{2}\xi }{dt^{2}}$$

where **n**—the normal to the surface, and ξ—mechanical displacement.

For the numerical solution of the differential Equation (1), in addition to the boundary conditions, it is necessary to use the initial conditions, which are: zero pressure and zero displacement in the material at the initial moment *t* = 0:

$$\begin{array}{l} p=0\\ \frac{\partial p}{\partial t}=0 \end{array}\}\begin{array}{l} \xi =0\\ \frac{\partial \xi }{\partial t}=0 \end{array}\}.$$

### 3.2. Model Specifications

**Figure 3:**
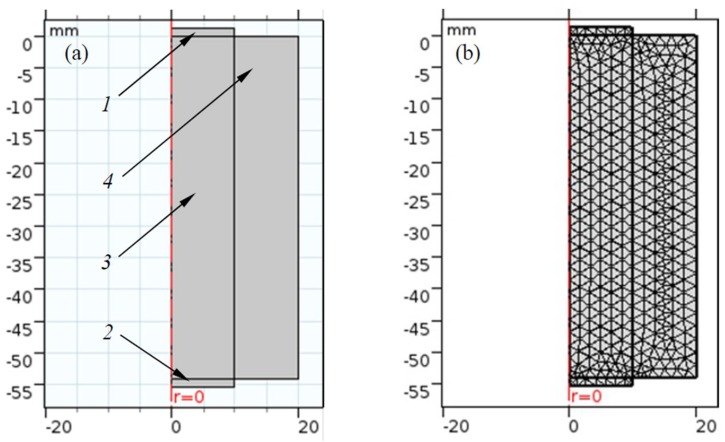
The studied geometrical area in the COMSOL Multiphysics environment: (**a**) two-dimensional (2D); (**b**) meshed simulation zone.

The geometry of the model created in COMSOL Multiphysics completely corresponds to the experimental one. Figure 3 shows this geometry. The ordinate axis coincides with the axis connecting the centers of the radiating and the receiving piezoelectric plates. The abscissa axis is for the radius (*r*) of this axisymmetric model. The radiating piezoceramic plate *1* is loaded on one side of the glycerin, and is excited by an electric pulse of a complex shape (Figure 2). The radiated acoustic pulse, after passing through the glycerin layer *3*, comes to the receiving piezoceramic plate *2*. The edges of both piezoelectric plates are rigidly fixed. The carrier frequency of the electric exciting pulses was 1 MHz, the thickness of the piezoceramic plates was 1.25 mm, and the diameter was 20 mm. The piezoelectric elements were coaxially located at a distance of 108 mm from each other. The material of the piezoelectric elements (piezoelectric ceramics PZT-19) had the following parameters:

$$\mathrm{density}\;\rho =7740\;\mathrm{kg}/m^{3}$$

$$\varepsilon =\left[\begin{array}{ccc} 1360 & 0 & 0\\ 0 & 1360 & 0\\ 0 & 0 & 1490 \end{array}\right]$$

$$s_{}^{E}=\left[\begin{array}{cccccc} 15.2 & -5.8 & -5.3 & 0 & 0 & 0\\ -5.8 & 15.2 & -5.3 & 0 & 0 & 0\\ -5.3 & -5.3 & 16.9 & 0 & 0 & 0\\ 0 & 0 & 0 & 42.6 & 0 & 0\\ 0 & 0 & 0 & 0 & 42.6 & 0\\ 0 & 0 & 0 & 0 & 0 & 21 \end{array}\right]\cdot 10^{-12},{\;\mathrm{Pa}}^{-1}$$

$$d=\left[\begin{array}{cccccc} 0 & 0 & 0 & 0 & 450 & 0\\ 0 & 0 & 0 & 450 & 0 & 0\\ -125 & -125 & 304 & 0 & 0 & 0 \end{array}\right]\cdot 10^{-12},\;C/N.$$

The finite element method is only applicable for finite geometric size models. A divergence of the ultrasonic beam causes possible reflections from the boundaries of the glycerin layer *3*. The calculation model uses a perfectly matched layer *4* to avoid re-reflections. This layer omits without reflection waves falling into it from other zones, and does not reflect them back [30].

The area subject to study was divided into sub-areas *dx* (Figure 3b), according to the following criterion:

dx=λ/16,

where λ is the wavelength in glycerin. Time step *dt* was selected according to the criterion Courant–Friedrichs–Lewy (CFL), which is a necessary condition for the stability of the numerical solution of the differential equation [31]. For the two-dimensional case, this criterion is as follows:

(2) $$c_{x}\frac{dt}{dx}+c_{y}\frac{dt}{dy}<\mathrm{CFL},$$

where $c_{x},c_{y}$ are the speeds of sound in glycerin along the axes *x* and *y* correspondingly; and *dx* and *dy* are the steps along axes *x* and *y*. Since glycerin is a linearly isotropic material, then , $c_{x}=c_{y}=c$, and CFL=1. Then, Equation (2) can be rewritten as follows:

$$2c\frac{dt}{dx}\leq 1\;\mathrm{or}\;dt\leq \frac{dx}{2c}.$$

As a result, considering that dx=λ/16 and c=λf, where *f* is the frequency of excitation, the time step selection criterion becomes the following:

$$dt\leq \frac{1}{32f}.$$

## 4. Comparison of Experimental Data with the Simulation Results

In Ref. [22], the estimated shapes of the electrical signals at the output of the receiver (by the d’Alembert method) are compared with the experimental data. Now, it is also of interest to compare the experimental data with the results of finite element simulation. For comparison, as in Ref. [22], we use a voltage at the surfaces of the receiver. The results of the present work in combination with the data presented in Ref. [22] can be useful to designers of piezoelectric transducers for applied acoustics problems.

**Figure 4:**
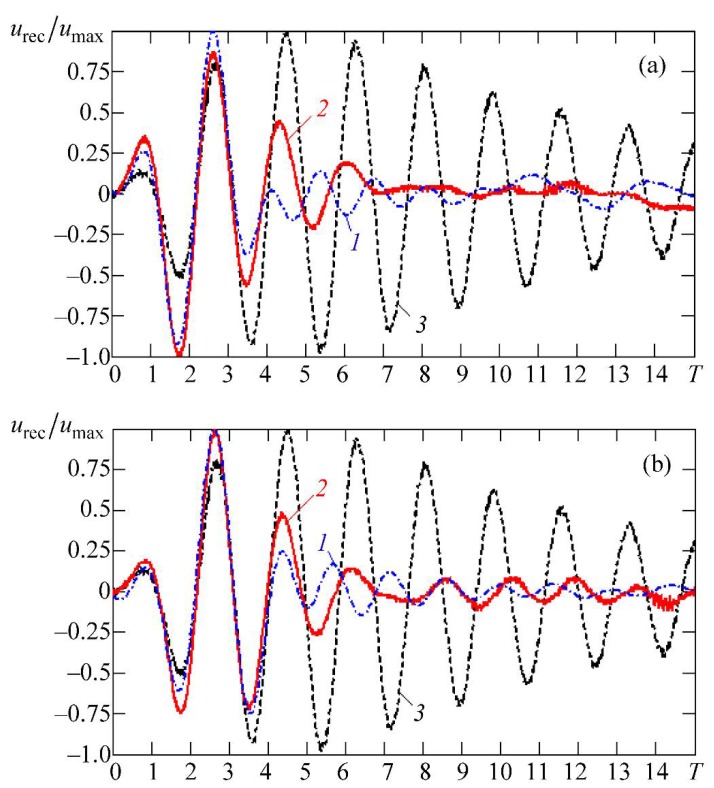
Shapes of electrical signals at the output of the: (**a**) shows the results when the emitter receives the signal shown in Figure 2b; (**b**) shows the results when the radiating plate is excited by the electrical pulse that is shown in Figure 2c.

Figure 4 shows some shapes of electrical signals at the output of the receiver. These shapes correspond to the electrical pulses that are applied to the radiator, as depicted in Figure 2a–c. In Figure 4, curve *1* corresponds to the calculated data obtained by the finite element method and curve *2* corresponds to the experimental data. Figure 4a shows the results when the emitter receives the signal shown in Figure 2b. Figure 4b shows the results when the radiating plate is excited by the electrical pulse that is shown in Figure 2c. In addition, Figure 4a,b contains one more curve: number *3*. It corresponds to the signal that is obtained experimentally at the output of the receiver when an electrical pulse excites the radiator, as shown in Figure 2a. All of the pulses presented in Figure 4 are normalized to the unit, i.e., normalized to the amplitudes of the signal maxima $u_{\max}$ for each of the received pulses $u_{\mathrm{rec}}$ that are carried out. The parameter $u_{\mathrm{rec}}/u_{\max}$ is a result of normalization, and is placed along the ordinate axis. On the *x*–axis is the dimensionless time $T=t/\left(T_{0}/2\right)$, where $T_{0}=1$ μs is the period of the signal at the natural frequency of the plate, and *t* represents the physical (real) time.

An analysis of the curves, as presented in Figure 4, indicates that curves *1* and *2* are very similar to each other. The main parts of the pulses are almost identical; some differences are observed only in the final (“tail”) part of the signal. One can see that the duration of the signal at the receiver output in the experiment (curve *2*) and in numerical simulation (curve *1*) is greatly reduced in comparison with the signal corresponding to curve *3*. For example, for the case presented in Figure 4a, it is reduced from 27 half-cycles to 11 half-cycles (−20 dB from the maximum value).

One can note that the “tails” of the pulses on curves *1* and *2* are distorted in frequency in comparison to the main (initial) part of the pulses. This can be explained by the influence of the compensating half-wave of the signal coming to the radiator.

In Ref. [22], an infinite plate was assumed as a model of a piezoelectric element to simulate by the d’Alembert method. The obtained numerical results were used as estimates, which were necessary for the analysis of transient processes in a finite-size piezoelectric element by COMSOL Multiphysics. Both considered methods give qualitative and quantitative results that are similar to the experiment.

## 5. Conclusions

Thus, the pulse mode of operation of the radiation-reception system consisting of two identical piezoceramic plates is studied numerically and experimentally. Glycerin was chosen as the immersion liquid. It was experimentally confirmed that the excitation of the radiator by a complex electric signal, the shape of which is determined theoretically and numerically, allows reducing the duration of the signal at the output of the receiver. The durations of the output signals were compared at the excitation of the radiator by different types of signals: by one half-wave of the natural frequency of the emitter, and by the signals of complex shape. The similarity of both forms of the calculated and experimental signals at the output of the system was noted.
